# Prevalence of diabetes mellitus in patients with newly evaluated papillary thyroid cancer

**DOI:** 10.1186/1756-6614-7-7

**Published:** 2014-09-11

**Authors:** Yannis M Paulus, Elyn R Riedel, Mona M Sabra, Robert Michael Tuttle, Marcia F Kalin

**Affiliations:** 1 Memorial Sloan-Kettering Cancer Center, Department of Medicine, 1275 York Avenue, New York, NY 10065, USA; 2 Memorial Sloan-Kettering Cancer Center, Department of Epidemiology and Biostatistics, 1275 York Avenue, Box 313, New York, NY 10065, USA

**Keywords:** Diabetes mellitus, Papillary thyroid cancer, NHANES, Risk factor

## Abstract

**Background:**

This study investigates whether diabetes mellitus is a risk factor for the development of papillary thyroid cancer, using an age-, gender-, and race-matched analysis.

**Methods:**

We retrospectively reviewed the charts of 1559 patients with newly evaluated thyroid cancer over a 4-year period at our institution and identified 1313 patients (84%) with papillary thyroid carcinoma. Characteristics of patients with diabetes versus those without diabetes were compared with a chi-square test for categorical variables and the Wilcoxon Rank Sum test for numeric variables. The prevalence of diabetes among patients with papillary thyroid carcinoma at our institution was compared (using an age-, gender-, and race-matched analysis) with that expected based on data from the continuous National Health and Nutrition Examination Survey (NHANES) from the same time period.

**Results:**

For patients with papillary thyroid carcinoma, the median age was 47 years; 74% were female; 83% were white; and the prevalence of diabetes was 8%. Among those with diabetes, 92% had type 2 diabetes, and 24% were treated with insulin. Risk factors for diabetes included age and race. The prevalence of diabetes among patients with papillary thyroid carcinoma of all ages versus that among patients from NHANES of all ages was not significantly different (RR 1.07, CI 0.88 - 1.28). The prevalence of diabetes among patients with papillary thyroid cancer who were 44 years of age or younger versus that among patients from NHANES who were 44 years of age or younger, however, was significantly increased (RR 2.32, CI 1.37 - 3.66). There was no significant difference when subgroup analysis was performed by gender or race.

**Conclusions:**

We found an increased prevalence of diabetes in patients with papillary thyroid carcinoma who were 44 years of age or younger.

## Background

Diabetes mellitus is a risk factor for cancer, specifically of breast, endometrium, bladder, liver, colorectum, and pancreas [1]. The pathophysiology of how diabetes contributes to cancer growth or development is an area of active investigation. The most commonly proposed mechanism is that hyperinsulinemia, which results as a compensatory response to the insulin resistance underlying type 2 diabetes, is mitogenic, through activation of the insulin receptor or the insulin-like growth factor-I receptor [2], which in turn stimulates cellular proliferation and inhibits cellular apoptosis [3]. Another possible mechanism is that hyperglycemia promotes carcinogenesis by increasing oxidative stress [1].

Several previous studies have tested for an association between diabetes and thyroid cancer, including one case control study and three large cohort studies. The case control study, which comprised 110 patients with anaplastic thyroid cancer, found a 4-fold increased prevalence of diabetes [4].

In the National Institutes of Health-AARP diet and health study, which involved a cohort of almost 500,000 adults, the prevalence of diabetes was 7.4% in women, and 10.1% in men. Over 10 years, 585 cases of thyroid cancer were identified. Women with diabetes had a 1.46 increased risk for all thyroid cancers, a 1.25 increased risk for papillary thyroid cancer, and a 1.92 increased risk for follicular thyroid cancer. There was no significant difference in the risk of thyroid cancer for men with diabetes or for the combined group of men and women with diabetes [5].

A cohort study of 4.5 million male veterans with 1053 cases of thyroid cancer found non-significant increases in risk for thyroid cancer among patients with diabetes: 1.2 for less than 5 years of diabetes, and 1.1 for at least 5 years of diabetes [6].

A population-based study of 1 million patients in Taiwan with 943 cases of thyroid cancer found no association between diabetes and thyroid cancer; less than 5 years of diabetes was associated with a decreased thyroid cancer risk [7]. In summary, the data from previous studies differ regarding a potential association between diabetes and thyroid cancer.

Our study is unique in that it is the only study to focus exclusively on patients with papillary thyroid carcinoma, the most common type of thyroid cancer. Using an age-, gender-, and race-matched analysis, we compared the prevalence of diabetes in patients with papillary thyroid carcinoma at our institution with that expected based on data from the continuous National Health and Nutrition Examination Survey (NHANES) [8] from the same time period.

## Methods

### Patient chart review

A retrospective chart review was performed after approval of an exemption from IRB/PB Review by the Memorial Sloan-Kettering Institutional Review Board/Privacy Board and in agreement with the principles of the Declaration of Helsinki involving research involving human subjects. A database was generated of all patients with newly evaluated thyroid cancer at one institution, Memorial Sloan-Kettering Cancer Center, for 4 years, from January 1, 2005, to December 31, 2008, totaling 1,559 patients. The database included patients with thyroid carcinoma newly diagnosed at our institution as well as patients with a prior diagnosis of thyroid carcinoma referred from the outside. All patients received a rigorous internal review of histology. Extracted data included age at diagnosis, gender, race, type of thyroid cancer, diagnosis of diabetes, and antidiabetic medication use at the time of papillary thyroid carcinoma evaluation. After a diagnosis of papillary thyroid carcinoma as proven by pathology was reached at our institution, a subsequent diagnosis or use of antidiabetic medications was not included.

Of the 1559 patients identified, 8 entries were duplicates; 17 patients had no primary thyroid cancer diagnosis; and 13 patients had unknown race. These 38 patients were excluded. We further restricted our cohort to those with papillary thyroid carcinoma and excluded 24 patients with anaplastic thyroid carcinoma, 40 patients with follicular thyroid carcinoma, 41 patients with Hurthle cell thyroid carcinoma, 49 patients with medullary thyroid carcinoma, 52 patients with poorly differentiated thyroid carcinoma, and 1 patient with minimally invasive hyalinizing trabecular carcinoma. Of the original 1559 patients, 1313 patients with papillary thyroid carcinoma remained for analysis (84%).

### Statistical analysis

Characteristics were compared between patients with diabetes and those without diabetes using the chi-square test for categorical variables and the Wilcoxon Rank Sum test for numeric variables. To determine whether the rate of diabetes was higher than expected in the group of patients with newly evaluated papillary thyroid carcinoma at our institution, we compared their observed prevalence of diabetes to the prevalence of diabetes in patients in the National Health and Nutrition Examination Survey (NHANES). NHANES is a federal program that seeks to assess the health and nutritional status of adults and children in the United States and has a publicly available data set on the Centers for Disease Control and Prevention website. We used NHANES data for 2005–2008. The expected number of patients with diabetes was estimated by multiplying the NHANES rate of diabetes for each age, sex, and race subgroup by the number of thyroid cancer patients within those subgroups. The observed and expected numbers of cases of diabetes were then separately summed and the relative risk (RR) was expressed as the ratio of observed-to-expected cases. The 95% confidence interval was calculated based on the assumption that the observed number of patients with diabetes was distributed as a Poisson variable.

We analyzed patients 44 years of age or younger separately from those 45 years of age or older based on literature suggesting that age 45 years or older is a risk factor for diabetes [9]. We also analyzed separately patients 65–74 years of age and those 75 years of age or older to separate elderly patients who practice healthy behaviors that enhance their life span and could be protective against diabetes [10].

## Results

### Patient characteristics

The demographics of the patients with papillary thyroid cancer in our cohort are listed in Table 1. The median age at evaluation was 47 years, with a range from 3 to 91 years of age; 74% were female; 83% were white; and 8% had diabetes. Of the 110 patients with diabetes, 92% had type 2 diabetes, and 6% had type 1 diabetes (Table 2); 24% of patients with diabetes were taking insulin.

**Table 1:** Patient characteristics

		**All patients N = 1313**
Age at evaluation	Median (range)	47 (3–91)
	0 - 44	589 (45%)
	45 - 64	535 (41%)
	65 - 74	143 (11%)
	75+	46 (3%)
Sex	Male	347 (26%)
	Female	966 (74%)
Race	White	1096 (83%)
	Black	50 (4%)
	Hispanic	72 (5%)
	Asian	86 (7%)
	Other	9 (1%)
Diabetes at evaluation of thyroid cancer	No	1203 (92%)
	Yes	110 (8%)

**Table 2:** Characteristics of the diabetes diagnosis at the time of initial thyroid cancer evaluation

		**Diabetes (n = 110)**
Type of diabetes	Type 1	6 (6%)
	Type 2	101 (92%)
	Secondary to steroid use	2 (2%)
	Unknown	1
On insulin	No	84 (76%)
	Yes	26 (24%)

### Prevalence of diabetes in patients with papillary thyroid cancer versus that from NHANES

Using an age-, gender-, and race-matched analysis as described in the methods, we compared the prevalence of diabetes in patients with newly evaluated papillary thyroid carcinoma at our institution versus that expected from NHANES data (Table 3). For patients of all ages with newly evaluated papillary thyroid carcinoma at our institution, there was a non-significant trend for a higher rate of diabetes (odds ratio 1.07, CI 0.88 - 1.28). For patients aged 0 – 44 years with newly evaluated papillary thyroid carcinoma at our institution, however, there was a statistically and clinically significant increase in the prevalence of diabetes, with more than twice the expected number of patients with diabetes (ratio 2.32, CI 1.37 - 3.66). There was no significant difference when subgroup analysis was performed with stratification based on gender or race.

**Table 3:** Prevalence of diabetes in patients with newly evaluated papillary thyroid carcinoma at our institution compared to prevalence expected from NHANES

		**Observed # of patients with diabetes**	**Expected # of patients with diabetes**	**Ratio**	**95% Confidence interval**
All patients		110	103	1.07	0.88-1.28
By age	0-44	18	8	**2.32**	1.37-3.66*
	45-64	61	59	1.03	0.79-1.32
	65-74	23	29	0.81	0.51-1.21
	75+	8	8	1.04	0.45-2.05
By sex	Male	31	31	0.99	0.67-1.41
	Female	79	72	1.10	0.87-1.37
By race	White	78	78	1.00	0.79-1.24
	Black	8	8	1.04	0.45-2.05
	Hispanic	10	9	1.10	0.53-2.02
	Other	14	8	1.74	0.95-2.91

*Indicates statistically significant difference.

### Risk factors for diabetes

The characteristics of patients with diabetes versus those without diabetes were compared (Table 4). Age was found to be a significant risk factor for diabetes, with patients aged 44 years or younger less likely to have diabetes, and an increasing frequency of diabetes with each subsequent age group. Gender was not a risk factor for diabetes, but race was a significant risk factor, with white race being protective for diabetes compared to other groups: 7% in whites, 16% in blacks, 14% in Hispanics.

**Table 4:** Risk factors for diagnosis of diabetes

		**Prevalence of diabetes (%)**	p-value*
Age at diagnosis of PTC	0-44	18 (3%)	<0.0001
	45-64	61 (11%)	
	65-74	23 (16%)	
	75+	8 (17%)	
Sex	Male	31 (9%)	0.65
	Female	79 (8%)	
Race	White	78 (7%)	0.003
	Black	8 (16%)	
	Hispanic	10 (14%)	
	Other **	14 (15%)	

*P-value calculated using age as a continuous variable.

**Asian and other race categories were combined to match how race is collected in NHANES.

## Discussion

Diabetes is an epidemic disease with extensive ramifications on various organ systems. The long-term complications of diabetes comprise not only the classically cited ones, including retinopathy, nephropathy, neuropathy, coronary heart disease, stroke, and peripheral vascular disease, but also cancer. Patients with diabetes have an increased risk of cancer of breast, endometrium, bladder, liver, colorectum, and pancreas [1].

Previous studies regarding the risk of thyroid cancer in patients with diabetes have yielded conflicting data. A case–control study showed a 4-fold increased prevalence of diabetes among 110 patients with anaplastic thyroid cancer [4]. One cohort study with 585 thyroid cancer cases showed an increased prevalence of thyroid cancer among women with diabetes but not among men with diabetes [5], whereas two other cohort studies, with 1053 [6] and 943 [7] thyroid cancer cases respectively, showed no significant increase in the prevalence of thyroid cancer among patients with diabetes.

Using a database of more than 1500 patients with thyroid cancer and an age-, gender-, and race-matched analysis, we compared the prevalence of diabetes in patients with newly evaluated papillary thyroid cancer with that expected based on NHANES data. For patients of all ages, we found a non-significant increase in the prevalence in diabetes. For patients 44 years of age or younger, we found a significant increase in the prevalence of diabetes that was more than twice expected.

This is the first study to focus on the prevalence of diabetes exclusively in patients with papillary thyroid carcinoma and the first study to demonstrate an age association between diabetes and thyroid cancer. Since the cancer was newly evaluated for inclusion in the study, the diagnosis of diabetes preceded the diagnosis of thyroid cancer, suggesting that diabetes was a risk factor for the papillary thyroid carcinoma in the subset of patients 44 years of age or younger. It is possible that the diagnosis of papillary thyroid carcinoma led to a diagnosis of diabetes by connecting young people to the healthcare system, thus accounting for a higher prevalence of diabetes compared with their peers. Akker et al. recently demonstrated an increase in insulin resistance in patients with differentiated thyroid cancer [11], however, consistent with the hypothesis that diabetes increases the risk of thyroid cancer through hyperinsulinemia and activation of the insulin receptor or the insulin-like growth factor-I receptor. Recent evidence suggests that activation of the mitogen-activated protein (MAP) kinase signaling pathway is important in the development of insulin resistance, type 2 diabetes, and cancer [12,13].

One limitation of our study is that it is retrospective, which exposes the study to bias, including selection bias of patients. Another limitation of our study is that our study population comprises newly evaluated thyroid cancer patients at a single, tertiary care referral institution over a four year period. Furthermore, our study population is predominantly Caucasian (83%). Finally, as there is no longitudinal follow-up of patients, there is no mechanism to determine the prevalence of diabetes after the diagnosis of thyroid cancer.

## Conclusions

Our study suggests that diabetes is a risk factor for papillary thyroid carcinoma in patients 44 years of age or younger. Further investigation is needed regarding the relationship between diabetes and thyroid cancer and their possible shared pathophysiology.

## Abbreviations

NHANES: National health and nutrition examination survey; RR: Relative risk; CI: Confidence interval; MAP: Mitogen-activated protein.

## Competing interests

The authors declare that they have no competing interests.

## Authors’ contribution

YMP, MFK, and RMT designed the initial study and prepared the final version of the manuscript. MFK supervised the conduction of the study. RMT provided the patient database. YMP reviewed the patient charts, acquired the patient data, and prepared the draft of the manuscript. ERR performed the statistical analysis and MMS assisted with analysis and interpretation of the data revised the manuscript. All of the authors critically revised, read, and approved the final manuscript for submission and agree to be accountable for all aspects relating to the accuracy and integrity of the work.
